# Patient Assessment and Chronic Pain Self-Management in Ethnomedicine: Seasonal and Ecosystemic Embodiment in Ayurvedic Patient-Centered Care

**DOI:** 10.3390/ijerph17082842

**Published:** 2020-04-21

**Authors:** Vinita Agarwal

**Affiliations:** Department of Communication, Fulton School of Liberal Arts, Salisbury University, Maryland, MA 21801, USA; vxagarwal@salisbury.edu; Tel.: +1-410-677-0083

**Keywords:** Ayurveda, chronic pain, self-management, ethnomedicine, environment, mind-body, ecosystems, geographical regions, Ayurvedic physician, patient-centered care

## Abstract

*Background:* Ayurveda’s preventive focus complements its strength with the interventionist approach of the biomedical in chronic pain self-management. Patient-centered care (PCC) using ethnomedicine promises greater patient self-management; however, few studies have examined environmental relationships and PCC in self-management of chronic pain through Ayurveda. *Objective:* To examine how Ayurveda’s philosophical focus on whole system frameworks describes the integration of the individual and the ecological in tailoring an integrative patient-centered diagnostic and prognostic approach to chronic pain management. *Methods:* This qualitative case study conducted in-depth semi-structured interviews of Ayurvedic physicians from India (*N* = 10) and a qualitative inductive content analytic approach. *Findings:* The diagnostic and interpretational framework of the *doshas* supports the integration of the individual and the ecological through (a) the circadian and seasonal cycles relating mind-body awareness with diet, lifestyle (e.g., yoga), and breath (e.g., *pranayama*)*,* and (b) biogeographical and ecosystemic regions relating the biogeographical and the ecological (e.g., *desh*) with the regulatory principle of pain and its physiological and anatomical perception (*vata*) in an approach that goes beyond treating pain etiology to a whole person PCC approach. *Conclusions:* The study highlights how circadian and seasonal cycles and evolutionary spatial-temporal factors of biogeographical and ecological regions are employed in patient assessment and self-management to support patient involvement. Recommendations for PCC in integrative chronic pain management include supporting patient ownership of their care through the *dosha* framework that relates the individual and the ecological in the patient’s own life-context and supports co-creation of a collaborative plan of care using an ethnomedical framework.

## 1. Introduction

Globally, about one in five people suffer from chronic pain [1]. Chronic pain is associated with opioid dependence, depression, disability, and reduced quality of life [2,3]. Studies show patient-centered care ((PCC); [4]) supports self-management of chronic pain [5,6]. PCC is emphasized in complementary and integrative medicine (CIM) therapeutic relationship where it is associated with self-management, provider guidance, and reduction in pain medication use in chronic care management [7,8]. However, PCC in CIM use is underexamined for chronic pain self-management despite evidence of its efficacy (e.g., in Traditional Chinese Medicine TCM, massage); [9,10,11]. Evidence suggesting Ayurveda’s multimodal approach comprising mind-body, nutrition, and yoga-based components may be efficacious in chronic pain domains (e.g., chronic musculoskeletal pain, [12]; neuropathic pain, [13]; ankylosing spondylitis, [14]; chronic low back pain, CLBP, [15,16]) has furthered recent interest in building an evidence base validating Ayurvedic approaches. This case study of Ayurvedic physicians’ chronic pain management protocol examines how Ayurveda as an ethnomedical system integrates an ecological sensibility in a tailored patient-centered approach. 

Chronic pain is a complex phenomenon comprising biological and psychosocial factors unique to each patient. However, there are methodological gaps for tailoring care in this domain thus, PCC use in chronic pain management has been suboptimal [17]. Chronic pain is one of the most cited reasons among individuals for using alternative therapies [18,19,20] and treating chronic pain syndromes [21,22], however the efficacy and mechanisms of CIM approaches including the role of therapeutic relationship and PCC for the treatment of chronic pain remain poorly understood and controversial [19]. 

Ayurveda is the traditional system of medicine following the scientific tradition of equilibrium and balance between man, health, and illness practiced in South Asia since about 3000 BC. The practice of Ayurveda is interrelated with the resources derived from the natural environment. Calls for integration of Ayurveda’s “multimodal, multidisciplinary therapeutic approaches such as meditation, exercise, patient education, and behavioral and psychosomatic therapies” in chronic pain condition management [23] (p. 3) have referenced the need to expand legitimization of distinct medical knowledge philosophies. For instance, in the biomedical approach, the source of health and disease is located in an identifiable objective position in the body. In contrast, the Ayurvedic approach utilizes distinct categories of diagnosis and prognosis (e.g., *dosha, agni, prakriti*) both pedagogically and professionally [24]. Such gaps in conceptual understandings of distinct medical systems risk the appropriation of practices (e.g., acupuncture or yoga) as unilateral biomedical empirical categories with a loss of contextualization in the philosophical and ontological knowledge base that furthered the diagnosis and prognosis protocols [24,25]. 

The Ayurvedic whole system approach categorizes chronic pain in various ways. For example, musculoskeletal pain arises from bones, joints, muscles, or soft tissues and is related with *vata* vitiation in the tissue (e.g., muscle tissue—*mamsa dhatu* or adipose tissue—*meda dhatu*), bone (e.g., *asthi*), or joints (*sandhi*), or during joint movements (e.g., knee synovitis-*kroshtukasheersha*), calcaneal spur (*vatakandakam*) or CLBP (*kaateesula*). Ayurveda also classifies pain by duration (e.g., acute-*ashukarisula*/chronic-*chirakarisula*, e.g., rheumatism due to blood disorders, *rakta—vata rakta;* or vitiated *vata* in joint/*sandhi*, e.g., osteoarthritis—*sandhigatavata*), site (e.g., localized-*ekangasula—*e.g., CLBP*/*generalized-*sarvangasula*, e.g., whole body pain), severity (e.g., superficial-*uttana*/deep-*gambeera*), predominance of *dosha* (e.g., *vakita, paittika, kaphaja, tridoshaja*), organ (e.g., visceral), and nature (e.g., slicing pain-*bheda—*e.g., heart disease*, hridayaroga*, profound pain-*avagadanja*, or cutting pain-*todahahulam;* [26]). Increasing the CIM evidence base in ethnomedical modalities such as Ayurveda that are employed in chronic pain management may lead to improved programs for patient education, care, and outcomes [27] through the integration of whole person approaches. 

However, the integration of Ayurvedic research in particular and whole system medicine research in general faces several barriers. While promising, the body of pain research in ethnomedicine is not consistent across modalities leading to challenges in its effective integration with conventional medicine in pain management [28]. Current research in Ayurveda has been critiqued as predominantly serving to extend the goals of conventional medicine rather than to validate its fundamental concepts using a whole system testing approach [29,30]. A major focus of existing studies has been on the reduction of objectively measured outcomes (e.g., pain interference, severity of disability). Investigators involved in Ayurvedic research face the challenge of conducting clinical research in abstract Ayurvedic concepts and principles such as *dosha, prakriti*, and *agni* [31] that are fundamentally distinct from and poorly understood in biomedical ontologies. However, some ethnomedical approaches have been rigorously investigated in clinical settings. Studies in acupuncture, for instance, have demonstrated how the meridians (Jingluo in Chinese), neurotransmitters, and endogenous substances may respond to needling stimulation and electrical acupuncture providing evidence of the neurobiological mechanisms of acupuncture in autonomic regulation [32]. Others, have examined the analgesic effects induced by acupuncture and moxibustion, including the actions of endogenous opioid and non-opioid mediated analgesic mechanisms as dependent on the frequency of afferent discharges activated by acupuncture and moxibustion stimulation [33]. Finally, the integration of Ayurveda in chronic pain management is complicated by a loss of variation in cultural, social, and religious beliefs as they inform its medical practices and philosophical principles.

Ethnomedical training in whole system approaches such as TCM and Ayurveda, in addition, require medical education and credentials that are equivalent in time and effort with biomedical education, such that completion of optimal physician training is often achieved in one or the other system. This bifurcation underscores the wide gap in the distinct philosophical approaches of ethnomedical and biomedical systems and highlights the need for studies explicating the epistemological approaches employed by different ethnomedical systems. As a primarily diet-and plant-based medical system, Ayurveda’s preventive focus, emphasis on health promotion, and self-management of chronic conditions complements its strength with the interventionist approach of the biomedical in chronic pain self-management with some evidence suggesting reduction in analgesic and cortisone usage [16]. 

Increasing recognition of the intimate connection of the health of ecosystems with the socio-cultural and health-based values of ethnomedicine [34,35,36] has led to calls for acknowledging ethnomedical practices with mutual respect in the push to further collaboration with biomedically-focused public health professionals [37]. Ayurvedic preparations constitute predominantly herbal and polyherbal plant-based ingredients selected based on ecological habitat (with the soil and water properties) and ecosystemic properties including seasons, climate and the harvesting, storage, and processing methods for their mechanism of action to stabilize the *doshas* [38]. Furthering understanding of the interrelationship of ethnomethodological perspectives and cultural behaviors, community values, and natural resources can situate the links between community empowerment, sociocultural determinants of health, and the environment [39,40] in an ecosystemic framework. 

The Ayurvedic approach as a medical system goes beyond its *materia medica* to positioning what it means to be human in relationship with the ecological in conceptualizing individual and universal health and relating the relevance of indigenous knowledge systems to environmental research. The Ayurvedic philosophy rests on a principle of equilibrium (*tridoshas, saptadhatus*, and *trimalas* in individual and planetary systems), nourished by the *jatharagni* (digestive strength) of the abdominal region, and metabolized through the processing of nutrients (*ahara rasa*) into the tissues (*dhatus*) for regeneration and nourishment by the tissue digestive/metabolic fire *(dhatavgni)*. The tissues progress sequentially (*rasa, rakta, mamsa, meda, asthi, majja*, *shukra*) from the subtle and tangible to the excretory portions (*sukshma, sthula*, and *mala bhaga*). Obstruction and vitiation in these processes is thought to lead to imbalance and, eventually, disease. Such an approach connects human health with planetary health through a multimodal approach emphasizing prevention, promotion, and personalized medicine within a daily and seasonal framework of diet, yoga, and mind-body-lifestyle integrations. 

Ayurvedic practices vary in their interpretation and application to account for the geographical or climatic region (e.g., coastal, marshy, or temperate) [41]. Ecological heterogeneity and biogeographical characteristics are spatially unique and vary with human, physical, and biological factors. They integrate elements such as cartography, climate, vegetation, land units, and biogeography in characterizing biodiversity. For instance, five major climatic types are recognized based on annual and monthly cycle averages of temperature and precipitation (e.g., tropical moist climates, dry climates, moist mid-latitude climates with mild or cold winters, or polar climates; Köppen Climate Classification System). Ayurveda’s contemporary development has been critiqued for its emphasis on pharmacological diagnostic and treatment approach on the one hand and shifting between biomedically-analogic terms like Ayurvedic biology, Ayurgenomics, and whole system clinical research on the other [41]. 

### Environment and Chronic Pain Management in Biomedicine

Recent biomedical theories of pain have begun to recognize the influence of environmental factors such as the patient’s social environment on adaptive responses to pain. Research in this direction has, in contrast, sought to understand the role of sensory stimuli from the environment in influencing the pain experience and the effect of lifestyle choices, such as meditation and yoga on reducing pain perception [42,43]. For instance, pain as a human feeling has been seen as akin to a sensation and a behavioral motivational drive similar to hunger and thirst [44]. Others find that recovery of surgical patients as assessed by shorter postoperative stays, fewer evaluative comments in nurse notes, and fewer potent analgesics was faster for those patients whose room had a window with a view of a natural setting in comparison with those whose room had a window facing a brick wall [45].

Likewise, earlier studies have demonstrated the therapeutic potential of visual stimulation as a nursing intervention in blocking off the anxiety-inducing sights and sounds of hospital surroundings and creating a pleasing environment [46]. In this case, the researchers find that subjects who were assigned to a soundless visual stimulus of a video display of natural scenery reported significantly greater pain tolerance (when pain reported as intolerable) and pain threshold (when detectable pain reported) than patients who were assigned to a display of a static blank screen. While studies have noted the effect of meditation on modulation of pain perceptions (pain intensity or unpleasantness); [47,48], other studies find a significant association between behavioral anger expression and momentary chronic pain intensity, with elevated behavioral anger expression linked to greater subsequent pain intensity [49]. 

The efficacy of clinical strategies integrating ethnomedical approaches (e.g., acupuncture, Ayurveda’s mind-body practices) in chronic pain management has been demonstrated in recent reviews [50]. However, chronic pain management can be challenging for clinicians without input from a multidisciplinary team that provides a range of perspectives and skills [51]. Evidence supports the efficacy of Ayurvedic medical practices in integrative chronic pain management, however gaps in understanding of the philosophical orientation of specific ethnomedical systems with a preventative and diet-based whole system focus like Ayurveda impede uniform integration [52]. This study furthers understanding of the Ayurvedic chronic pain management protocol through its examination of how Ayurvedic physicians’ philosophical focus on whole system frameworks describes the individual and the ecological in tailoring an integrative patient-centered approach to chronic pain management. 

## 2. Methods

### 2.1. Participants and Procedures

The qualitative case study focused on purposive sample of expert participants who were Ayurvedic physicians in India with a Bachelors of Ayurvedic Medical Science (BAMS) degree recruited from a professional center of training and practitioners identified by public searches based on purposeful maximum variation (Table 1). Participant recruitment (*N* = 10) for the study was concluded based on data saturation in a tightly focused content domain with low variability, once sufficient depth and breadth of information and perspectives were identified with ongoing preliminary analyses during the data gathering process [53]. Participants were recruited from a city in the south-west and one from the north-west region of India. Inclusion criteria included completion of recognized degree in Ayurvedic medicine and being currently in practice or teaching of the modality as an Ayurvedic physician for greater than the past year. Interviews were audio-recorded as in-person interviews or interviews conducted over Skype (audio only) and transcribed by a professional transcriptionist company. 

A semi-structured in-depth interviewing protocol (Appendix A) was employed to explicate participant beliefs, thoughts, and practices. The researcher completed in-person guided training in diet and nutrition principles from Ayurveda experts in India. Her analytic approach supports the case study methodology as it is imbued with the observations, interactions, and communicative constitution of health alongside the practices, culture, and philosophy through her immersive experience in the field. The case study method was selected to elucidate conceptual dimensions of a less understood, but tightly identified conceptual domain and because of its close alignment with the study goal to enhance trustworthiness [54,55]. The researcher observed the practice in situ in India in provider offices in major and regional urban centers, official tours of national and international Ayurvedic medical institutions (e.g., pharmacological laboratories and plants), and of artifacts in natural surroundings in urban and rural centers in India. Practices in public (e.g., treatments advertised in shops, claims by individual vendors) were observed (Table 2 provides a summary of research methodology flow). 

### 2.2. Ethical Considerations

IRB approval (Human Subjects Review Committee, Salisbury University, Maryland, USA*,* FWA00020237) for the study protocol (Protocol # 52) was received on 29 April 2019, for the larger project examining Ayurvedic mind-body therapies in chronic pain management with a specific interest in understanding the practices employed by practitioners, the philosophical and cognitive-affective-physical context, use of communication to evoke relaxation or modulate patient attention, and mode of delivery. Participants received a copy of the informed consent form electronically for their records. Informed consent was obtained through oral administration of the informed consent form to participants (audio-recorded) prior to participation in the study. 

### 2.3. Case Study Approach Employing Inductive Qualitative Content Analysis

This case study reports findings from a section of the data [55]. Inductive qualitative content analysis was employed for data analysis [56]. In the first pass through the data, the researcher conducted a close reading of the different concepts and processes followed by the Ayurvedic physicians in managing chronic pain, paying attention to the description of the holistic philosophy as it informed their understanding of pain and considerations of the patient’s diagnosis and treatment plan. Because the knowledge domain of Ayurveda emphasizes the operation of individual factors in a synergistic relationship with mechanisms unique to the patient, the condition, and the environment, the content analysis recognized that the responses were abstracting knowledge addressing a niche area of focus of this paper. The researcher made memos of the primary concepts in Ayurvedic physician discourse. In the second pass, close attention was paid to the primary focus of the present study, the construction of the chronic pain management protocol in relationship with the environment, and understanding Ayurvedic medicine as a whole system ethnomedical approach. In this stage, a line-by-line process of open coding highlighting key concepts under the questions from the interview protocol that probed the role of *desh* (physical or geographical place) and chronic pain management, and of the questions that probed the conceptualization of cycles (e.g., daily and seasonal or *dinacharya* and *ritucharya*) and chronic pain management was conducted. Subsequent passes through the data helped identify axial codes that comprised the descriptive themes (e.g., how did the physicians’ descriptions connect cycles with pain management protocols, how was the external environment related with lifestyle in the pain management diagnostic process). 

**Figure 1:**
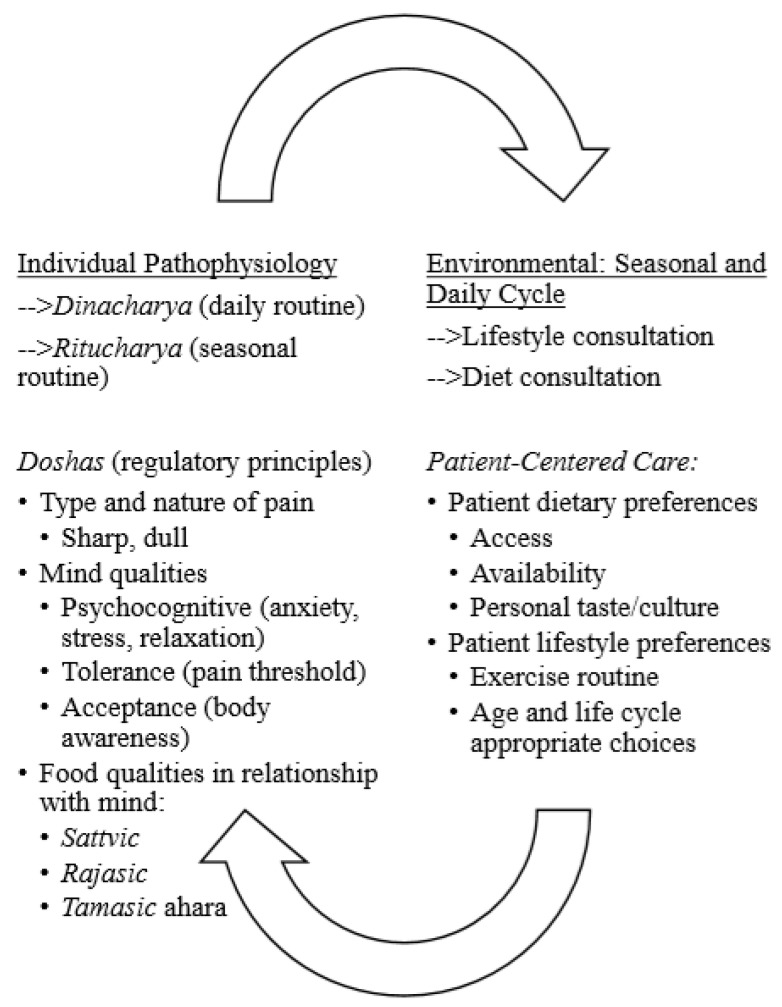
Patient-Centered Care in Ayurvedic Chronic Pain Management Protocol: Integrating Seasonal and Daily Cycles in Chronic Pain.

**Figure 2:**
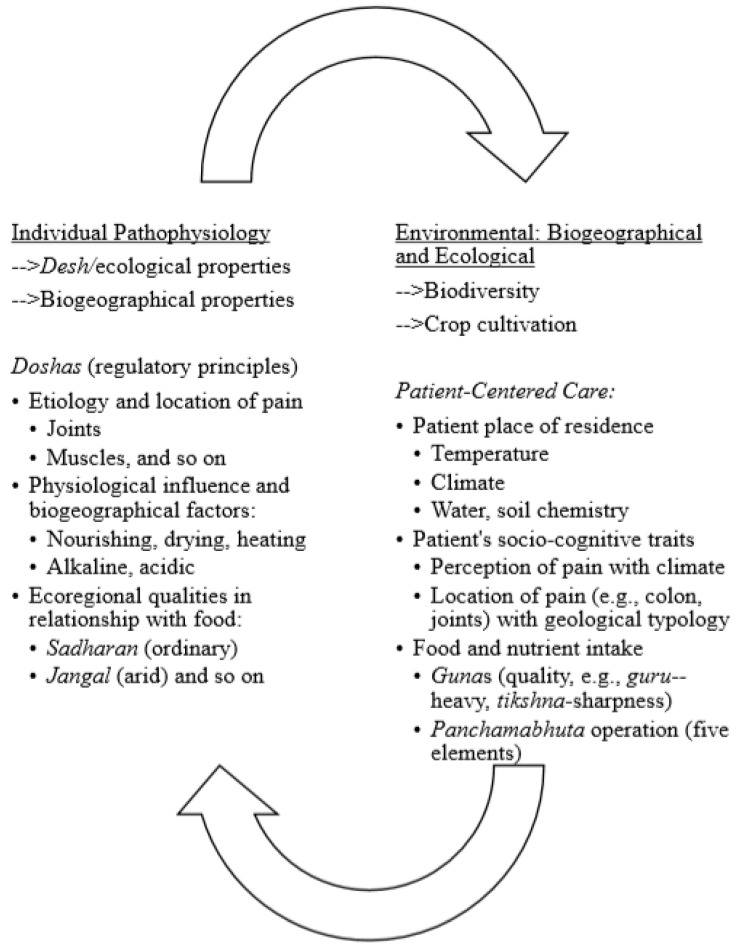
Patient-Centered Care in Ayurvedic Chronic Pain Management Protocol: Integrating Biogeographical and Ecological Regions.

In parsing second level analytical, axial codes, the “patterns, insights, and concepts” [55] (p. 167) that emerged in the descriptive level were identified (e.g., relationship with mind and qualities of food, nature and regulatory principles of the physiological body, or the *guna*s and *dosha*s, with the properties of the external environment, or *desh*) to understand and interpret relationships seen in the data. The themes were further collapsed through the process of abstraction and constant comparison in the case study (i.e., comparing codes and their underlying concepts) [57] to illustrate the inductively refined goal and findings via visual displays (e.g., figures), [55]; refer to Figure 1 and Figure 2. The themes in the findings identify core categories keeping interpretation close to participant descriptions and allowing the reader to corroborate the themes as they emerged from the interview process [56]. 

### 2.4. Validity and Reliability

Member validation was obtained through soliciting feedback from participants on the preliminary data analysis themes identified in the present study. As many of the concepts referenced had nuanced, culturally and philosophically-specific meanings, to maintain accuracy of representation and interpretation, care was taken to balance the analytic narrative with multiple participant voices. This practice allows the researcher to connect the practices followed by Ayurvedic physicians with the considerations involving the environment in the management of chronic pain. To minimize researcher bias and to enhance trustworthiness, multiple perspectives including the content (e.g., direct representation of Ayurvedic physician voice) and its relationship with the interview protocol are presented [53]. The researcher’s self-reflexivity was maintained through explication of the researcher’s positionality during the interview process and during the data analysis process. As a case study approach, the researcher undertook multiple experiential modes of approaching the phenomenon of inquiry [55] including a 10-day silent meditation retreat to further enhance her life-long experiential learning. 

## 3. Findings

An inductive qualitative content analysis of the interview texts explicating how Ayurvedic physicians’ focus on whole system approach considers the philosophical integration of the individual and the ecological identified the two themes (a) seasonal and daily cycles (Figure 1) and (b) biogeographical and ecosystemic regions (Figure 2) shaping subjective pain perception in tailoring a patient-centered approach to chronic pain management.

### 3.1. Seasonal and Daily Cycles with Subjective Pain Perception 

In the first theme, the emphasis on seasonal cycles (e.g., summer and winter) and quotidian cycles (e.g., of day and night) were identified in the Ayurvedic physician’s tailored chronic pain management protocol (refer to Figure 1). The seasonal and daily cycles are connected with the individual anatomical and physiological composition through the three regulatory control factors for the fundamental physiological processes that maintain their integrity through the biological history [16], called *doshas* (*vata, pitta,* and *kapha*), that comprise each and whose balance and aggravation shapes the individual pain perception. The seasons and daily cycles are connected with the food and lifestyle, each of which is governed by the *tri-doshas*. 

In patient consultation, the Ayurvedic physician considered the relationship of seasons with each individual patient’s unique lifestyle and situates this to understand the effect of daily rhythms on their pathophysiology. Physician A said that in devising a treatment plan for chronic pain, he will look at the “*dinacharya*, along with that diet … exercise … *yogasana* … *pranayama*, which is directly related with that joint pain, or … chronic pain.” *Dinacharya*, or the daily routine, alongside diet, exercise, as the *yogic asanas*, and *pranayama,* or the exercises pertaining to the breath, were emphasized in A’s response in designing an individualized protocol. Participant C emphasized these relationships: “apart from just giving medications or giving therapies, we, on the first day of consultation itself … It’s an intense consultation where we try to understand the lifestyle of the person, the kind of food the person is eating and even the kind of job, the kind of relation they have in the family and if that... What are the factors which may aggravate *vata*, which may aggravate stress and ultimately create more *vata.*”

The Ayurveda physician conceptualized individual agency as the mind, through the breath, and the regulatory principles, through the *tri-doshas* of the *vata, pitta*, and *kapha*, as key to their particular manifestation and operation in the individual body. Participant B described the role of *dinacharya* or daily rhythm with the *dosha*s (regulatory principles) in diagnosis and treatment of chronic pain: “At the beginning of the day, *kapha* is dominant, in the middle of the day … *pitta* is dominant, and at that end *vata* is dominant … If the pain is aggravating in the middle of the day, say, abdominal pain is aggravating in the middle of the day” understood in relationship with location and nature (e.g., sharp, dull, burning) of the pain: “some patients have … burning sensation, and they wake up because of that pain and burning sensation in their abdomen” (Participant B). Participant I explained how the Ayurvedic approach is based “on the sun, like *praat kaal* … or the sunrise time medicine, that is the *rasayan kaal”* tailoring treatment with the circadian rhythm: *“*Medicines given in that period … We have just had the bowel movements … so these medicines work, or the herbs work more efficiently.” Explaining how the day is divided into different sections, each governed by a particular *dosha*, Participant I said: “this is the morning, *kapha kaal*. Then comes the *pitta kaal*, that is 10:00 a.m. to 2:00 p.m. Then again, comes the *vata kaal*. So, it depends on the *kaal* also, that is the *vata, pitta, kapha* also, and the sun also.” Diagnosis and prognosis take the *doshas* into account with the solar movement in the sky: “Suppose you give medicine at 10:00 a.m. … so 10:00 a.m. the *pitta kaal* starts. If you give the medicines [then], it will work on the *pitta* or the *rakta*. If a person is suffering from acidity, or if he has some blood related problems, if you give medicines at 10:00 a.m., they will surely work very well.” However, the same medicine can have a different effect based on the circadian cycle and the individual patient: “Suppose on *mann* (mind) … then we give it mostly on the *udaan kaal*. It works on the *prayatna* (individual effort)*, oorja* (mental strength), and during *swapna kaal* (time of sleep/dreams) that is just before bed” (Participant I). 

Likewise, Participant B said: “we ask many other things, like, in which season this pain is aggravating, in which part of the day or night it is aggravating. Accordingly, we can come to some conclusion.” The same diagnosis will differ in its prognosis based on “for example, if this pain is aggravating in, say, rainy season when clouds come, so it is said that sometimes *ama* plus *vata, vata*, which is having some *ama* in it, that is undigested *vata*, that is aggravating in this rainy season when clouds come.” Accordingly, the Ayurvedic physician will attend to the balancing of *vata* and *pitta* through an examination of their relationship with the patient’s own *doshic* composition. The physician will also take into account the influence of the season on the natural elements, such as on the quality of natural water (heavy during the monsoons). 

For the Ayurvedic physician, the seasonal routines (*ritucharya)*, are in an intimate relationship with the individual human physiology and the *doshas*. Participant B described the role of seasonal and daily cycles in diagnosis and management of chronic pain thus: “if this pain is increasing only in cold seasons, then this pain is increasing because of *vata*, but that may not be [in] summer, and I will treat accordingly.” In other words, “When, in which season, in which part of the day or night this pain is increasing, we are to understand that, then diagnose and treat accordingly” (Participant B). The day is divided into eight sections, each governed by one of the *tridoshas* (*vata, pitta*, *kapha)*. These practices illustrate the relationship between the environmental rhythms and individual pathophysiology in pain perception and management. 

In determining the influence of the season or the day on the individual patient’s physiological perception of pain, the Ayurvedic physician considers diet as an important in the diagnosis and prognosis protocol. As Participant A describes, food is intimately connected with the mind and the body “because our *ahara* (food) creates our body. Whatever our body, it’s created by our *ahara*, whatever we are taking, it converts it into our body. Our food (*ahara*) nourishes our mind.” Thus, food comprises the body in a manner that the individual’s body, psycho-cognitive qualities (e.g., *sattvic, rajasic*, or tamasic) is made up of their daily diet, or *ahara*. As Participant A elaborated, “*sattvic ahara*” is food that nourishes the higher, reflective, contemplative, and thoughtful qualities of the mind. Likewise, *rajasic* and *tamasic ahara* nourish the active (outward agency) and the lower or less contemplative qualities of the mind.” By determining the dietary choices, the physician seeks to gain an understanding of the subjective experience of pain perception by the patient. 

In Participant C’s approach to treating patients with chronic pain, lifestyle is part of the intake interview: “if there are certain things which are *vata* increasing, for example, too much exercise. Too much physical exercise the body can’t handle.” In this case, by looking at age, “for example in 65 age, doing excessive exercise where the body’s *kapha* is not handling, plus he is skipping the meals, not having breakfast for example.” The diet is tailored to the patients: “If he likes to have drinks or beverages, then I can suggest some *khajur manthan*, dates smoothie.” As Participant C notes, “half of our time goes more into this. And it happens every follow-up. Because once you suggest, we have to see whether the person is following.” Monitoring and encouraging self-management is a key part of the Ayurvedic approach. For instance, if their patients are “not following, what are the challenges. I even go to the extent of understanding, if they are working in some pantry, what are the food available there, what can be the better choices … So, it goes to such a minute level of holding hands with the patient.” This attention to detail references the patient-centered and individualized approach of Ayurvedic medicine to the lifestyle-based management of chronic pain. 

In the first theme, *seasonal and daily cycles with subjective pain perception*, the Ayurvedic physicians connecting the cycles with the breath as the state of mind, and mind-body exercise (e.g., yoga), in designing the chronic pain management protocol for the individual patient. In tailoring the protocol, the physicians described the relationship of the balance or aggravation of the *vata, pitta*, and *kapha* during the seasons, particular time of day, and the food with the patients’ own unique physiological composition of the *tri-doshas* (Figure 1).

### 3.2. Biogeographical and Ecological Regions with Subjective Pain Perception 

In the second theme, the emphasis on biogeographical and ecosystemic regions was identified in the Ayurvedic physician's tailored chronic pain management protocol. Both biogeographical and ecological regions reference the processes that determine spatial and temporal patterns in nature, however biogeographical studies reference planetary evolutionary patterns while ecological studies reference adaptive evolutionary patterns in local areas [58]. The ecosystemic view accounts for flow and cycling in ecological systems without looking at population dynamics or organism activities [59]. Biogeography describes the evolutionary, climatic, and geological processes that explain the distribution of diversity on the planet. Analytical approaches related to mapping (e.g., geographical information systems), spatial relationships, and large-scale patterns in macroecology have allowed for greater inferential understandings. Ecology focuses on adaptive evolution, population processes, and abiotic and species interactions underlying distributions of species in local areas (i.e., within restricted spatial and temporal scales) and relies upon experimentation and statistical models for its inferential power [58]. As an ethnomedical approach, Ayurveda considers food, climate, and stress as potential disruptors of the normal regulatory principles governing the body beyond the cutaneous level [16]. The Ayurvedic physician’s description connects the biogeographical and the ecoregional with human health (refer to Figure 2). 

In examining a patient, the Ayurvedic physician considers diet based on the ecoregion, for instance, in their description of the “*desh ahara*, which means, [giving an example of the city the provider lives in] is the *sadharan* (ordinary, not extremely hot or cold) *desh*” (Participant A; see also primary Ayurvedic text, *Charaka Samhita*). As A describes, “when you are going to the coastal region ... in that region, salt is much more present in the environment also, in the water also, in the food also. In that case, we are giving them mainly the alkaline nature of the food.” Biogeographical examinations seek to understand how and to what effect the ecosystems and species distributed in a geographic space and geological time influence each other [60]. The physicians’ description illustrates how they take into account ecosystemic elements such as biota (e.g., invertebrates for lakes, wetlands, and rivers), geological formations (e.g., sedimentary, metamorphic, or volcanic rock), soil (e.g., calcareous sands, clay-rich soil, leached soil, or brown forest soil), and categories (e.g., salt marshes, desert, coral reef) in assessing the individual patient’s physiology in chronic pain management. 

Through examining the interplay of the operational principles of the human body and the ecoregions (e.g., *dosha*s, *guna*s, *agni*), the theme of biogeographical regions suggests how these are understood as mutually influencing each other in the diagnosis and prognosis process for chronic pain management. In Participant A’s description, water chemistry (e.g., alkaline or acidic) and temperature is taken into account as the seeks to “balance the pH, means [when] the environment is acidic in nature, and we are giving the alkaline food. It balances the pH of the body, and it give nourishment to the body.” Balancing soil characteristics and food with the geographical region, A explains: “if you are in north part of the India …where too much cold is present in the environment, ice is present all over the environment.” These are associated with the biodiversity distribution and vegetation qualities that correspond with the regulatory principles. In his example above, A describes how, “in that condition, we are using the *sarson* … [or] *methi* (mustard). It is hot in potency.” Here *hot* refers not to the objective temperature but to the abstract mechanism through which mustard operates in the human body and is inferred through the foundational texts that recognize the properties of biotic and abiotic elements will evolve over the succeeding centuries. 

In other words, the ecoregions are associated with the *tridoshic* energy dimensions that interact in particular ways with the individual’s own composition of the energy dimensions. As B describes: “The places where *vata* is more, like arid lands, the dry desert lands. In such places, the same patient, if he’s staying in, say, more medium … atmosphere and if that patient goes to this such place, his pain may aggravate. *Desh* [ecoregion] also plays important role in pain.” In other words, “In the *jangal* land or arid land, the dryness is more, more many times” (Participant B). Because dryness is one characteristic of *vata*, its ecoregional aggravation will act in particular ways with the patient’s own physiological pain perception (the perception of pain is governed by the *vata* principle) and its distribution that can then be shaped by the diet, soil characteristics, water, and other ecological elements of the region that reflect the particular energy composition of that ecosystem and geological typology. 

The climate regions (comprising factors such as latitude, solar radiation, air mass, pressure zones, oceanic heat exchange, mountains, winds, and altitude) shape the pain experience in the patient. As Participant B explained, “it may be winter; it may be hot season. Every time the dryness is more … the nourishment and dryness is lost from the place [in the body], the joint, so this condition will worsen in such situations if he moves to that place, or otherwise he moves to cold places, the *vata* has cold quality.” Such reasoning underlies the Ayurvedic physicians’ recommendation to support the alignment of particular ecoregions in different chronic pain diagnoses and individual energy dimensions. As illustrated in B’s assessment of chronic pain management: “*vata*, it aggravates in cold places, so this person if moves to cold areas also, then also the pain is going to aggravate, so you have to take care of such things.” 

In the case of chronic knee pain, with the complex array of factors including the cartilage, ligaments, tendons, and menisci, associated with degenerative tissue disorders (e.g., osteoarthritis), auto-inflammatory diseases (e.g., rheumatoid arthritis) the underlying premise, as B explained, was: “if the environment is dry…the dryness will increase in your body also, in the person’s body also. That is affecting the knee also.” For Participant C, “in diagnosis we take into [account] … ten factors and that includes *desh* … If a person is living in Bay area with so much *vata* aggravation. Every hour there is a change in … climate itself [which] defines more *vata*. So, there the protocol will be [s]trict, more important for *vata* disease for example.” By examining the variation in the biogeographical factors with the individual’s anatomical and physiological factors and regulatory principles, this illuminates how the ecoregions are intimately connected with chronic pain diagnosis and treatment in the domain of chronic knee pain. 

An example of how an Ayurvedic practitioner connects the factors in their treatment management approach of osteoarthritis by examining the functioning of the digestive system is illustrated by Participant C: “If the cause of pain in the joints, be it osteoarthritis, if the main cause is more gases in the colons. Now Ayurveda connects that the *vata* aggravation is primarily at the level of colons.” In Ayurveda, each organ has its own role in balancing the doshas. Thus, as C explains: “I have to first clean the colon. I have to give some *panchakarma* to cleanse it, detox it. Then I have to lubricate the colons, so that there are no more *vata* generation there.” Once the *vata* generation has been balanced in the colon, C “will suggest food which will not create more *vata* because of the food as the source of more gas or *vata* aggravation. I will suggest food which will create more lubrication, more *kapha* enhancement to pacify *vata.”* Thus, for C, the osteoarthritis pain management protocol will involve, “a good cleanse and strengthening of colons. Second thing, diet. Third thing, [the] connection between the mind and intestine.” C addressees the mind and the intestine with the osteoarthritis pain management protocol by applying the Ayurvedic principle which “says these are the two places of *vata*, mind and colons … So, if the person is in too much *chintan* (rumination) and *chinta* (worry). I have to deal there also … And fourth, then I will give some herbs.” In sum, C will “find the exact cause where the *vata* is aggravating. Is it only at the mind, is it only at the colons or is it at the extremities level, where more physical work and [is] it more related to *indriya* (sense organs)?”

Biogeographical regions represent discrete homogenous areas wherein natural communities and species interact with the physical elements of the environment and are employed to model patterns in biodiversity. Participant H clarified how the conceptualization of the geographical place with ecoregion and biogeographical elements shaped the chronic pain management approach: “So many times I think *desh* is like the location where the person is staying … if the person is staying in all places, definitely their inflammation or pain increase and if you’re staying in some hot places, then pain would be less.” Conversely, “in the marshy places … where there is very less sunlight, can also increase the pain in the [p]atients.” Participant I elaborated: “With *ahar* (food) and *vihar* (place), at least *desh* (ecoregion) is related with the [flow of *vata* energy in the body, *vata*].” If the energy flow “is in *apana kaksha*, then the *ahar* will also depend on the pain, which will not increase in the *apana kaksha* (downward regions).” Accordingly, Participant I stated, “treatment will also differ according to the *desh* … suppose you have a knee joint pain, then you will have to prescribe … medicines, which will be given before the meals. That is, which works on the *apana kaksha* … considering the *desh* (ecoregion) as a body … as a *kshetra* (place) … [and as] … related to areas also.” The nature of the pain varies with the ecoregion and the treatment will consider *guna* (quality) pertaining to the five elements (*panchamahabhuta*): “the herbs will be different. We have to use more *ushna* (heat), *tikshna* (sharpness) … The intensity of the *guna*s, like *ushna, guru* (heavy), *snigdha* (unctuousness) will be more.” In Participant I’s description, the patient’s body is considered an ecoregion with analogous characteristics to the external environment and is assessed during chronic pain diagnosis and manifestation. 

In the second theme, *biogeographical and ecological regions with subjective pain perception*, the Ayurvedic physicians described the consideration of unique ecoregional and biogeographical elements (through *desh*) in relationship with the energy dimension of pain and its physiological and anatomical perception (*vata;* Figure 2). In tailoring care, the physicians described the relationship of the quality of the abiotic and biotic elements of the environment (e.g., acidic or alkaline soil) with the *virya* (hot or cold potency) of vegetation consumed as food (e.g., mustard). Thus, the diagnosis and prognosis is tailored to each individual and is wholistic in its approach by taking into consideration its relationship with the ecological and biogeographical elements and systems in designing the chronic pain management protocol. 

## 4. Discussion

Ayurvedic physicians integrate the individual and the ecosystemic by emphasizing seasonal and daily cycles and biogeographical and ecological regions to understand subjective pain perception. Relating seasonal and daily cycles in chronic pain management supports PCC by facilitating mindfulness of the patient’s daily routine set within seasonal cycles, and connecting them with the cycles of the breath, or the mind, and yogic exercise, or the body, in a tailored process. Relating biogeographical and ecological regions in chronic pain management supports PCC by facilitating mindfulness of the biogeographical and ecological regions and connecting them with their biotic and abiotic characteristics through the *dosha* regulatory principles shared by the individual and the ecosystemic. The chronic pain management protocol connects the ecosystemic with the individual through *vata* as the regulatory principle that governs the body’s chronic pain perception and connects the psychosomatic (e.g., stress, anxiety, relaxation) with the pain domain (e.g., musculoskeletal, neuropathic; [4]). 

The study identifies how the individual-environmental (im)balance was assessed by the Ayurvedic physician during patient intake through *dosha*s, quality of the mind (through the *pranayama*), and lifestyle in relationship with the daily and seasonal cycles and biogeographical and ecological regions to tailor the pain management protocol in ways that empower the patient (Figure 1). Integrative pain management approaches could benefit from a consideration of the patient’s subjective pain experience of the pain situated within a framework of lifestyle, diet, meditation, and exercise to conceptualize health in an organic perspective. The relationship between health and environment in Ayurvedic chronic pain management is embodied through seasonal and daily cycles and biogeographical and ecoregional spatial-temporality with the regulatory principles of the *tridosha*s. The universal/individual intersection of *dosha*s in this theme situates the patient as an autonomous individual contributing to an interactive shared decision-making approach in the pain management protocol. The concept of renewal and regeneration is embedded in the seasonal and circadian rhythms to mutually constitute the human body with the environment in a harmonious relationship. In this relationship, the cycles reference the *vata, pitta*, and *kapha* functions as regulating principles that govern pain qualities. The anatomical and physiological body of the patient is considered analogous with the body of the biogeographical and ecological region, through their embodiment of the *dosha*s and their mutually-shaping action on each other. These elements are assessed by the Ayurvedic physician in relationship with the etiology and pathology of chronic pain in the patient. 

Situating the patient within a spatial proximal (ecological) and distal (biogeographic) environment embeds environmental awareness in constructing a patient-centered approach to chronic pain management and supports active patient involvement by constructing a collaborative physician-patient partnership grounded in mutual exploration and understanding of the pain domain and its subjective perception. Evidence suggests patients prefer “partnership, participation, deliberation/negotiation, doctor knowledge/recommendations, mutual agreement…information exchange, and flexibility” [61] (p. 210) in the physician-patient relationship. Embedded in the Ayurvedic physician's protocol is the assessment of how environmental disruption and imbalance uniquely influences the balance and regulation of the constitutive elements of their patient’s body (Figure 2). The *dosha* regulatory principles are the governing factors responsible for specific functions during the course of the individual’s lifespan such as input/output processes and movement (*vata*), metabolism and energy production and turnover (*pitta*), and storage, structure, and lubrication (*kapha*). In the chronic pain condition, the physicians emphasized the understanding of the *vata* principle, and their diagnosis and prognosis process took into account the relationship between seasons, daily routine, place of residence and origin, and climate in determining the operational mechanisms of activating nociceptive and neuropathic pain the pain management plan. Such a plan was in alignment with the individual’s physiological regulatory processes and the external environment such as soil quality, humidity, temperature, acidity/alkalinity, food choices, and circadian rhythms. 

Viewing chronic pain management as an intricate relationship of each individual patient’s diet and lifestyle in relationship with the ecosystemic elements illuminates how the Ayurvedic physician seeks to understand how the cycles relate routine with food (*ahara*) to align quotidian rhythms with the patient’s practices and yoga *asana*s. The qualities (*guna*s) of the food support specific qualities of the mind and its physiological attributes. The Ayurvedic physicians’ discourse notes the influence of cycles (seasonal, quotidian, life-term) and geographical place (*desh*) in supporting patient-centered chronic pain management at both sensory and affective levels. Explanation and connection of seasonal and quotidian cycles with diet and its metabolic mechanisms in the body illustrates to the patient how their pain behaviors (e.g., withdrawal, escape) are related with affective aspects (e.g., nagging, discomfort, excruciating) and their psychosocial expressions (e.g., anxiety, depression, stress). 

For the Ayurvedic physician, the body is nourished by food, or *ahara* it consumes, constituting its very nature (*guna*s, such as *sattva, rajas*, and *tamas*). The processing of the food into its different qualities shifts with its production, consumption, and assimilation. Food, in its connection with biogeographical and ecoregional qualities, reflects and integrates the biogeographical *dosha* principles. In this patient-centered assessment, the Ayurvedic physician’s pain management practices integrate the seasonal variations (*ritucharya*) in the lived context of the patient. By communicating advice from the patient's own lived routine and environmental context, the Ayurvedic physician helps support the patient in taking ownership and control of their chronic pain management.

### 4.1. Limitations

As a small qualitative case study, the findings are interpretive and descriptive in nature, drawing upon observation and explanation. As the researcher conducted the data analysis, member validation of preliminary themes allowed for strengthening the internal validity, transferability, and credibility of the findings [56]. Adopting a multi-modal view to data collection and an experiential methodological approach yielded focused insights that illuminate the complexity of the issue [62]. The study makes a significant contribution by illuminating novel insights highlighting the relationship of the seasonal rhythms and ecosystemic environment in patient-centered integrative pain management and suggesting directions for future research on sustainability (e.g., in modifications to local biota and climate impacting Ayurveda’s plant-based pharmacopoeia) to connect with pain behaviors and their psychosomatic elements alongside cultural and relational factors. Future investigations can further understandings of how ethnomedical practices reflect the unique vulnerabilities of the relationship of human health with the environment in their integration of geographical properties in constituting wholeness [63].

### 4.2. Pragmatic Recommendations

To support the consideration of psychosocial factors alongside the clinical syndromes of chronic pain the study recommends providers: (a) include the consideration of biogeographical and ecoregional place to integrate the influence of the lived environment in the diagnosis and treatment of chronic pain. For instance, in the provider-patient encounter, providers can explore the geographical context of the patient’s place of residence alongside their diet choices in ways that integrate their *dosha* principles with local produce to better guide their patient’s ownership of their pain experience; (b) educate patients about the influence of the ecosystemic environment on the body’s metabolic and anabolic processes, such as by encouraging knowledge of the acidic or alkaline soil properties on the digestive and metabolic process, consumption of locally sourced food, awareness of temperature and climate, the qualities of their nutrition (*ahara*), Such provider communication enhances the goals of integrative patient-centered care by supporting patient ownership of their chronic pain management; and, (c) support holistic understandings of pain in relationship with lifestyle for the patient, particularly as it is impacted by seasonal and circadian rhythms. Such care centers the patient’s lived environment and cultivates a whole person approach to chronic pain management. 

## 5. Conclusions

This qualitative case study contributes to the call for chronic pain management to go beyond a focus on treating pain etiology by envisaging a patient-centered whole person approach (body, mind, spirit). The findings identify how the *dosha* regulatory principles serve as a diagnostic and interpretational framework in chronic pain management by relating the individual (circadian and seasonal) and ecosystemic (biogeographical and ecological regions). As shared regulatory principles operating at both individual and the environmental levels, the *dosha*s facilitate patient ownership and control through patient education and application in their life-context to co-create a collaborative patient-centered plan of care. Pragmatic recommendations for integrative chronic pain management highlight how circadian and seasonal cycles and long-term evolutionary and local spatial-temporal factors can be employed in patient assessment and chronic pain self-management to support patient involvement and further the goals of patient-centered care. The study contributes to furthering understandings of how an ecosystemic approach to integrative patient-centered chronic pain management can center patient autonomy in furthering a collaborative decision-making approach in an ethnomedical framework. 

## Figures and Tables

**Table 1:** Participant Characteristics.

Pseudonym	Age (Years)	Education	Specialty	Profession *	Employment (Years) ***	City ^†^
A	32	MD Ayurveda ^‡^ (BAMS)	Dravyaguna	Ayurvedic Physician & Practitioner	9	Pune
B	45	MA, Ayurveda; MA, Sanskrit		Ayurvedic physician	20	Pune
C	44	BAMS & MD, Ayurved	Kayachikitsa	Ayurvedic physician	18	Pune
D	51	BAMS Ayurveda MS in Ayurvedic Dietetics	Medicine & Surgery	Ayurveda physician/ practitioner	27	Mumbai
E	33	BAMS Ayurveda	Medicine & Surgery	Ayurveda physician/ practitioner	10	Mumbai
F	26	BAMS Ayurveda	Medicine & Surgery	Ayurveda physician/ practitioner	2	Delhi
G	34	MD, Ayurveda	Charak Samhita	Ayurveda physician and academician	17	Pune
H	46	BAMS Ayurveda	Medicine & Surgery	Ayurved Acharya ** (Physician)	20	Pune
I	46	BAMS Ayurveda, MA, Yoga	Medicine & Surgery, Yoga	Ayurved Consultant, Physician, Yoga teacher	22	Pune
J	69	MD, PhD, Ayurveda	Medicine & Surgery	Professor & Government of India	35	Delhi

^†^ Location of current practice reported or where participant was based for a major duration. ^‡^ Ayurveda is used interchangeably with Ayurved and Ayurvedic. * Profession as self-described by participant. ** Ayurved Acharya references the Hindi translation for Ayurved Physician. *** Aggregate reported in cases where participants have had multiple concurrent or additional professional roles (e.g., Ayurvedic physician and yoga teacher or academician).

**Table 2:** Summary of Research Design Methodology Flow.

Phase I	Location	Phase II (Supporting Phase I)	Location	Goal	Outcome
Preliminary research examining current literature	USA	Review of author’s own previous findings and data	USA	Identifying conceptual domains, challenges, gaps	Semi-structured interview Protocol development (conceptual domains)
Vipassana meditation	Dhamma Giri, Igatpuri, Nashik, Maharashtra, India	Observation of cultural artifacts, symbolism, practices as they constitute healing, body-self integration, reflexivity, and whole-person conceptualization	Igatpuri, Shani Shingnapur, Nashik, Pune, New Delhi, India.	Experiential integration of body awareness, healing, reflection/reflexivity and whole-person conceptualization	Protocol construction, refining of conceptual domains and probes
Participant recruitment	Pune, Maharashtra, India and New Delhi, India	Advanced course in Ayurvedic Diet and Nutrition Tour of Ayurveda pharmacological laboratories, national and regional teaching institutions	Pune, Maharashtra, India	Understand Ayurvedic diagnostic and prognostic principles in chronic pain through deeper learning supported by semi-structured in-depth interviews with Ayurvedic physician participants	Recruitment of Ayurvedic physicians, scheduling interviews, administering informed consent, data transcription
Validity and Reliability/Rigor	USA and India	Data analysis and presentation	USA	Organization of data, member validation, iterative interpretation	Preliminary themes, saturation, fixing themes
Discussion and Synthesis of results	USA	Conclusion and recommendations	USA	Situating findings and contribution with prior studies, significance, and recommendations	conceptual framework, significance, future directions

## Appendix A

### Interview Protocol Domains (Partial List)

- Communication of mind-body-spirit practices for chronic pain
  - Ayurvedic protocol for increasing patient knowledge of their own body and managing pain with particular attention to patient *dinacharya/ritucharya/dosha/phanchabhuta/guna/veerya/vipaka*?
  - The *manas/atma* element in Ayurvedic practices. Role of the patient and *kal* in treatment.
  - Ayurveda’s description of the self (*atman*)-principle and its role in diagnosis and treatment in chronic pain. Mechanisms of operation of effective processes, identifying expected outcomes keeping in mind patient role and provider communication.
  - Understanding the mind (*manas*) in experience (*pragya*) in addressing pain related conditions. Understanding the principle guiding the use of herbs (*jadi/buti*) with mind (*manas*) in chronic pain management.
- CAM practice protocol: Communicating the connection between diet (*ahar) a*nd mind (*manas)* with respect to chronic pain management in the Ayurvedic protocol. Understanding the positioning of the location (*desha*)/environment in diagnosis and treatment relationship.
- Focusing attention (*man*) and regulating emotions in mind-body practices: Understanding the role and principle of operation of *ahar* and *vanaspati* (herbs) in this cycle/relationship in the Ayurvedic protocol.
